# From Concept to Clinical Practice: A Review of Autotransplantation Techniques and Their Impact on Dentistry

**DOI:** 10.7759/cureus.66904

**Published:** 2024-08-14

**Authors:** S Vandana Ajay, Rozina Vishnani

**Affiliations:** 1 Department of Oral and Maxillofacial Surgery, Sharad Pawar Dental College and Hospital, Datta Meghe Institute of Higher Education and Research, Wardha, IND

**Keywords:** tooth replantation, surgical retreatment, root development, avulsion fracture, tooth autotransplantation

## Abstract

The goal of autotransplantation of teeth (ATT) is to provide the patient with a functioning tooth to replace a missing one. In dentistry, this surgery has gained significant approval and popularity; nonetheless, there is still a shortage of thorough evidence about its long-term effects. Tooth transplantation has a rich historical pedigree, and the main factors determining its success are the extra-alveolar period, proper splinting, periodontal ligament treatment, and root growth stage. With its high reported survival rate, autotransplantation is a potential therapeutic option, especially when it comes to replacing damaged anterior maxillary teeth.

Collaboration between orthodontists, pediatric dentists, restorative dentists, and oral and maxillofacial surgeons is necessary for the successful execution of this treatment. The extra-alveolar period, proper splinting, periodontal ligament treatment, and the stage of root growth are the main factors that determine success. Although there are many applications for autotransplantation, a good functional and cosmetic result depends on careful patient selection and a proper surgical approach.

It is not practical to replace lost teeth in children and teenagers with bridgework or implants as this may interfere with the proper development of the alveolar process and other facial bones. As such, these techniques are not recommended. Alternatively, implanting a tooth from the same person without fully forming its roots might be a good substitute. This method promotes improved mastication, speech, dentofacial development, aesthetics, and arch form integrity by enabling unhindered alveolar growth and root development.

Although tooth autotransplantation has not been widely used in clinical dentistry, it is currently seen as a viable option that can replace traditional prosthetics and implant rehabilitation in both financial and medical terms. This review examines several benefits, possible iatrogenic harms, side effects, and important variables that might affect the result of the transplant, in addition to suitable criteria for the best-case selection. It also offers recommendations based on the literature.

## Introduction and background

An extracted or embedded tooth is transferred from one part of the patient's mouth to another, such as an extraction site or surgically created socket, in a dental surgical procedure known as autotransplantation [1]. In several cases, such as those involving impacted or ectopic teeth, early or traumatized tooth loss, tumor- or iatrogenic tooth loss, congenitally missing teeth, replacement of teeth with a poor prognosis, and developmental abnormalities when a suitable donor tooth is available, this procedure is advised [2,3].

There is still an unbroken recipient region that may be utilized for an implant, even if the transplant fails later. However, a detailed understanding of the variables affecting the long-term success rate is key for this approach. When applied correctly, this technique might be a useful addition to current therapeutic practice or even a stand-alone therapy alternative. According to dental literature, since impacted maxillary canines are so crucial to dentofacial aesthetics, they are often chosen for transplanting. Also, it is possible to implant a growing mandibular wisdom tooth into the socket of a first mandibular molar [4,5].

By providing the required proprioceptive stimulation, the transplantation of a third molar can also help to preserve alveolar bone volume and ridge architecture, minimize root resorption, and maintain natural spacing [2,6,7]. In young patients with developing alveolar bone, bridgework and Osseointegrated implants are generally contraindicated because the failure of the implant to erupt like a natural tooth in alveolar bone typically results in infra occlusion. Lost teeth in teenage patients are generally replaced by transplantation [6].

The stage of root completion, the kind of donor and recipient tooth, the way the tooth is handled during surgery, the recipient site's preparation, the use of systemic antibiotics during the healing process, and adjunctive procedures such as ex vivo root canal therapy (RCT), and the kind and length of splinting all have an impact on the prognosis [8]. This review aims to provide current and cumulative information on these prognostic indicators, which may contribute to reducing autotransplantation of teeth (ATT)-related problems and failures [8]. Thus, the goal of this in-depth narrative analysis is to offer dentists contemporary, evidence-based guidance that explores the history, specific treatment approaches, and anticipated results of tooth autotransplantation, surgical extrusion, and purposeful replantation procedures.

## Review

History

Tooth transplanting has been a part of human civilization for a long time. Renowned transplant researcher John Hunter documented a successful tooth allo transplant in London in 1772 [9]. Still, the majority of these early cases did not yield satisfying results. The first clinically accomplished successful autotransplantation was not until the 1950s, proving that carious first molars could be successfully replaced with immature third molars [10]. A case was published by Slagsvold and Bjercke, which is noteworthy. They put 34 premolars in place between May 1959 and January 1970 and monitored the results for an average of 6.2 years [11] Of note, 291 autotransplanted teeth - 121 of which were premolars - were implanted between 1955 and 1980, as documented by Schwartz et al. in 1985. They discovered that 56.6% of all tooth kinds survived after 10 years. On the other hand, autotransplanted premolars had a substantially higher 10-year survival rate than 75% as shown in a study [12]. They concluded that treatment using premolars that have both completely grown and growing roots is predictable when it comes to transplantation. A few years later, Andreasen et al. studied a larger sample size of autotransplanted teeth. The scientists used a standardized, non-traumatic surgical approach to track 370 autotransplanted premolars for 10-13 years [13]. They demonstrated that 98% and 95% of the mature and immature transplanted teeth made it through the process. Following the 23 immature and 22 mature teeth of 40 patients for up to four years, Kugelberg et al.12 discovered that 82% and 96% of the patients were successful [14]. After 50 successive autotransplanted teeth, over an average of 7.5 years. In their publication, Czochrowska et al. (2013) detailed the long-term outcomes of 30 autotransplanted teeth that were monitored for a duration of 17-41 years (average follow-up: 26.4 years), revealing success and survival rates ranging from 79% to 90% [15].

Indication

The four following clinical situations may warrant consideration of autotransplantation [16]: (1) trauma or pathology-related loss of one or more upper front teeth; (2) autotransplanting teeth that are ectopically positioned back into the same arch in the proper location, e.g., autotransplantation can be used to realign ectopic canines in situations when orthodontic alignment and traditional surgical exposure are not appropriate; (3) developmental abnormalities of teeth and related syndromes: regional orthodontia, tooth aplasia, cleidocranial dysplasia [5], and tooth agenesis [17] are examples of developmental defects of the teeth and related disorders that are indicative of the need for transplantation; (4) replacing lost anterior teeth may provide a challenging issue, which is especially concerning in youngsters whose face and jaws are expanding and whose dentition is still forming; children may have oral trauma or congenital hypodontia as the cause of their anterior tooth loss, which occurs just before the pubertal growth spurt and the formation of the permanent dentition.

Contraindications

Heart abnormalities, inadequate oral hygiene, low self-motivation, and lack of soft tissue to achieve primary site closure are among the contraindications. Resorption of the alveolar ridge may happen if the recipient site does not have enough Bucco palatal or buccolingual breadth to support the donor tooth [5]. If transplantation is delayed, it should be completed as quickly as possible in the next two months to avoid bone resorption compromising the donor tooth's wound bed during that time.

The technique of autotransplantation shows minimal variation, but the clinical protocols, systemic antibiotics, and local procedures to enhance viability varied among the primary studies included in the reviews. If the root resorbs and the root attachment diminishes, the tooth may become loose and eventually fall out. Maintaining good oral hygiene is crucial to prevent attachment loss, which can lead to treatment failure [6]. In cases of an infected site, autotransplantation cannot be performed. Additionally, the infraocclusal position of the transplant may require further interventions such as orthodontic and/or prosthodontic treatment. If the pulpal tissues fail to heal, endodontic treatment would be necessary.

Basic principles

The efficient healing depends on the “periodontal ligament” (PDL) being repaired, regardless of the age or immaturity of the teeth. While adult teeth cannot undergo pulp regeneration, immature teeth can. In instances of tooth autotransplantation, comparable healing processes are also discernible. Furthermore, bone induction may be an added advantage of autotransplantation. The next discussion will address PDL repair, bone induction, pulp healing, and root growth, among other elements of wound healing in autotransplantation.

Surgical procedure

The tooth that needs to be extracted from the receiving site must be extracted before the donor's tooth while performing an emergency tooth transplant. Evaluating the size, form, and condition of the PDL of the donor tooth is crucial. It's critical to keep the PDL safe. An intra-crevicular incision is made before luxation to preserve as much PDL on the root as possible, and the donor's tooth is extracted with the least amount of force and gentleness possible. The donor tooth must be replaced in its natural socket following extraction.

The tooth should be kept in a storage media that will preserve the viability of the periodontal ligament cells, such as Hank's balanced salt solution, if any extra-oral duration is anticipated. The primary ingredients of Hanks' Balanced Salt Solution (HBSS) are inorganic salts, glucose or pyruvate for energy, phenol red for pH indication, and sodium bicarbonate for pH stabilization1. The precise makeup of 1 milliliter is as follows: sodium chloride (NaCl); potassium chloride (KCl); calcium chloride dihydrate (CaCl_2_·2H_2_O); hexahydrate magnesium chloride (MgCl_2_·6H_2_O); trihydrate sodium acetate (C_2_H_3_NaO_2_·3H_2_O); dihydrate sodium citrate (C_6_H_5_Na_3_O_7_·2H_2_O). To change pH, use sodium hydroxide or hydrochloric acid; water for two injections [7,15]

Measurements are taken of the donor root's length and the mesiodistal breadth of the root and crown. The recipient socket is created marginally larger than the donor socket using surgical round burs that are run at a low pace and chilled with saline to provide antibiotics. The tooth that has to be retrieved from the receiving location should be taken out before the donor's tooth in an instantaneous transplant. The donor tooth has to have its PDL condition, size, and anatomical shape assessed.

It is important to avoid harming the PDL. For this procedure, it's critical to apply orthodontic force to the donor tooth four weeks before the surgery. This orthodontic force helps prepare the tooth for the upcoming surgery and ensures better outcomes. It is an important step in the overall process and should not be overlooked. After being extracted, the donor tooth needs to be reinserted into its original socket. Measurements are taken of the donor root's length and the media-distal breadth of the root and crown. With little speed and saline cooling, the recipient sockets were made somewhat bigger than the donor sockets with the use of surgical round bars. 

Autogenous tooth transplantation, which involves the relocation of a tooth from one site to another within the same individual, presents itself as a viable therapeutic option for managing severe cases of ectopic tooth eruption that would otherwise necessitate extraction. Advancements in 3D additive manufacturing technology play a pivotal role in this process by enabling the creation of a new recipient socket using a surgical replica of the tooth slated for transplantation. This innovative approach serves to streamline the procedure, potentially reducing the overall handling and extraoral time involved.

Healing

PDL Healing

There are two possible ways that the PDL cells might be harmed: either mechanically during the extraction procedure or biochemically as a result of different situations that occur outside of the mouth. These cells are especially prone to damage in stressful conditions such as pH variations, osmotic pressure changes, and dehydration [18-21]. We may expect a successful healing of the PDL if donor teeth are properly removed to avoid damage to the PDL and then stored in ideal circumstances outside the mouth until the conclusion of the surgical process.

Re-implanting an avulsed tooth into its original socket as soon as possible promotes the best possible healing of the PDL. Similarly, substantial PDL recovery is expected, albeit with somewhat less predictability, when a donor's tooth is inserted right away into a recipient socket that has just been pulled [18,22]. In contrast to the previously described circumstances, the healing process takes longer, and the prognosis is slightly worse when the donor's tooth is inserted into a freshly created (artificial) socket [22]. Although viable cells on the root surface are essential for complete healing, these variations in prognosis suggest that progenitor cells on the socket wall should not be underestimated [18].

Bone Healing

Bone is rapidly renewed throughout the bone induction process, and the lamina dura around the implanted tooth develops. It is observed that even in situations when the gap is large, bone graft materials are not required between the transplant roots and bone walls [23]. Nevertheless, if donor teeth are implanted at a recipient site with inadequate Bucco-lingual spacing and the roots protrude through a bone dehiscence. When it comes to this operation, the existence of bone induction surrounding a transplanted tooth offers a substantial benefit over employing implants.

Advantages

There are various benefits to this process. Compared to alternative techniques like dental implants or prostheses, autotransplanted teeth can assist preserve normal oral function and aesthetics. Second, the tooth is removed from the patient's mouth with biological compatibility guaranteed, removing the possibility of rejection and the requirement for intricate biocompatibility calculations. Furthermore, because autotransplantation eliminates the need for specific procedures and materials, it is frequently less expensive than prosthetics or dental implants [6,23].

Using a natural tooth also lessens the chance of bone loss, which can happen with dental implants, and preserves the surrounding bone structure. Furthermore, the process is occasionally simpler than implant implantation, which calls for further measures like sinus lifts or bone grafting. The amount of time the patient spends without a working tooth can be decreased if the implanted tooth integrates properly and starts to function right away.

One significant advantage is that when the PDL is intact, the tooth can continue to erupt. This physiological process also gives the clinician the ability to move the tooth using orthodontic forces, providing greater treatment flexibility. Finally, compared to certain prosthetic solutions, autotransplanted teeth can blend in perfectly with the surrounding natural teeth, providing outstanding aesthetic outcomes. To guarantee success and reduce any potential difficulties, a qualified dental practitioner must carefully design and carry out this surgery.

Complications

It is critical to comprehend the possible dangers and difficulties associated with tooth autotransplantation. One of the frequently encountered issues in dental health is tooth ankylosis, which is a condition where the tooth becomes fused to the surrounding bone. Even if the process can be quite successful, it's important to be completely aware of any potential problems. These include the transplanted tooth's rejection or failure, infection, periodontal problems such as surrounding bone resorption, reddening of the gingiva, ankylosis of teeth, bleeding, insufficient root growth, misalignment or bite problems, harm to the surrounding structures, the requirement for further procedures, worries about aesthetics, and the possibility of root resorption.

Planning carefully, using good surgical skills, and providing post-operative care are essential to reducing these risks and guaranteeing the procedure's success.

Other treatment options

There are various treatment alternatives to take into account when deciding how to replace or fix broken or missing teeth as shown in Table 1.

**Table 1:** Alternative treatment options for missing teeth

	Studies	Survival rate	Follow-up
Single tooth implant	Systematic review - Jung et al. [24]	94.5%	5 years
Fixed partial dentures	Systematic review - Lang et al. [25]	89.1%	10 years
Resin-bonded bridges	Systematic review - Pjetursson et al. [26]	87.5%	5 years

Dental implants, dental bridges, dentures, resin-bonded bridges, orthodontic treatment, gum and bone grafts, endodontic therapy (also known as root canal therapy), and orthodontic treatment are among these alternatives. Speaking with a dental expert is recommended as each choice has pros and downsides of its own. The optimal course of action will be determined by the patient's unique requirements, dental health, and preferences.

Further developments

To prevent issues with rejection, bioengineered teeth created in vitro using patient stem cells may be used in dental transplants in the future [27]. Currently, there is a restricted use of autotransplants since donor teeth are scarce. In principle, xenotransplants might bridge the "gap" in donor tooth availability, particularly for individuals with moderate to severe hypodontia. However, there are significant ethical concerns associated with the use of xenotransplants. If bioengineered or xenotransplanted teeth are ever recommended, 3D prototyping will help analyze the donor location and produce surgical templates to increase the procedure's likelihood of success.

Miscellaneous factors

Wet storage in a physiological solution and maintaining a minimum extraoral period have been demonstrated to be essential for pulp and PDL cell viability. If the tooth is kept dry for longer than thirty minutes, irreversible cell damage has been documented [13]. Many medical professionals advised keeping the samples in a sterile physiological saline solution and recommending an extraoral storage period of no more than 15 minutes [18,28,29].

For the graft to grow a longer root than those put in a more superficial, more occlusal location, it must be positioned at the same occlusal level as the donor site. In contrast, to minimize postoperative damage, the transplanted tooth should be positioned somewhat below the occlusal level if it has a mature root and has completely erupted [5,30]. The patient should be counseled to follow a soft diet in the first several days following the transplant.

Only three to six months following transplantation, according to some experts, it is found that [17,29] transplanted teeth can be subjected to orthodontic therapy. Several research studies indicate that to minimize the risk of ankylosis (the fusion of the tooth root with the surrounding bone), it is recommended to perform tooth movement at intervals of four to eight weeks. This approach is suggested to help prevent the occurrence of ankylosis during orthodontic treatment. Though it typically causes a slight increase in the frequency of surface and inflammatory root resorption, orthodontic treatment can be started as soon as the periodontal space regenerates and further confirmation of the lamina dura on the radiographs, according to Hamamoto and others [30].

## Conclusions

The field of autotransplantation has seen significant advancements in the past decade, with high success rates reported in various studies. When teeth are lost, autotransplantation is frequently not thought of as a treatment option. This is unfortunate since, besides being a very successful treatment modality with significant time and cost advantages over implants, the biological reasons for its success are recognized and the appropriate indications are often present. From the standpoint of the patient, a real tooth, as opposed to a mechanical prosthesis, preserves the dentition. Without a doubt, the dentist should be qualified to advise and perform this treatment on the right patient. Based on research and the latest advancements in dental technology, autotransplantation of teeth can provide patients with long-term satisfaction and healthy smiles.
